# Supplementary material for: Single-Cell Transcriptomic Profiling in Inherited Retinal Degeneration Reveals Distinct Metabolic Pathways in Rod and Cone Photoreceptors
Source: Int J Mol Sci. 2022 Oct 12;23(20):12170. doi: 10.3390/ijms232012170 (PMC9603353; doi:10.3390/ijms232012170)
Supplement: Supplementary file 1 [file ijms-23-12170-s001.zip › ijms-1914586-supplementary.pdf]

Supplementary data and figures

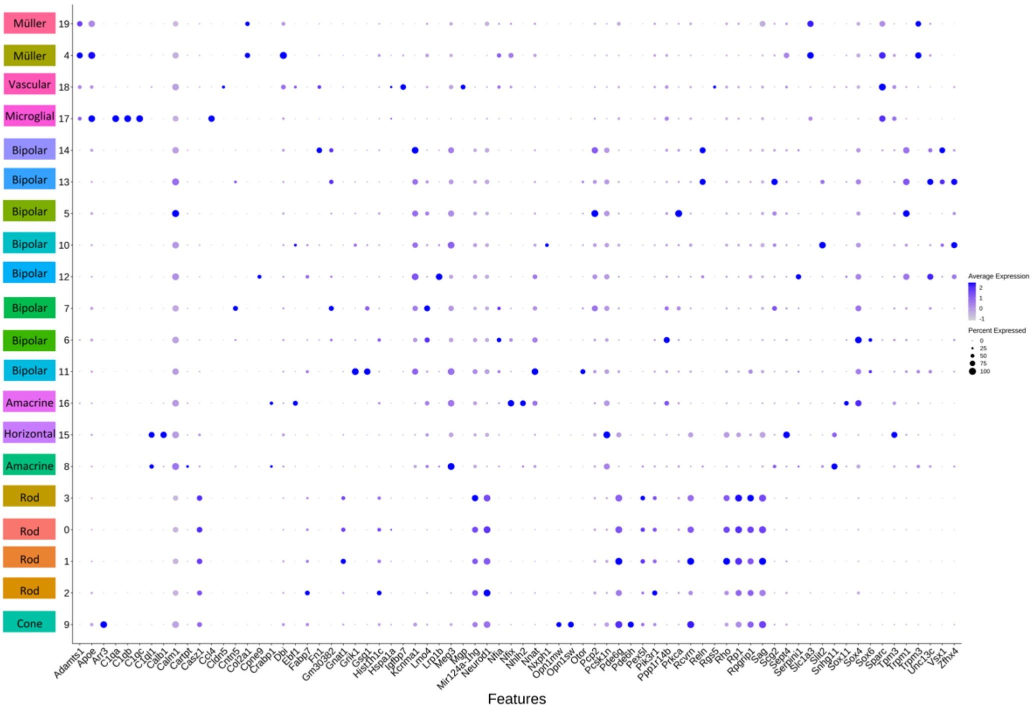

Figure S1. **Dot plot of the top four markers for each gene cluster.** The Seurat FindMaker function was used to analyze the differences between each cell population. Marker genes were selected based on an |avg_log$_2$FC| > 0.58 and a *p*-value < 0.05. Genes are plotted on the x-axis, the y-axis indicates the cluster ID. The cell types related to a given cluster are shown on the left. The color ramp indicates the average expression of a certain gene in cell population. Dot size indicates the percentage of cells expressing the gene in that population.

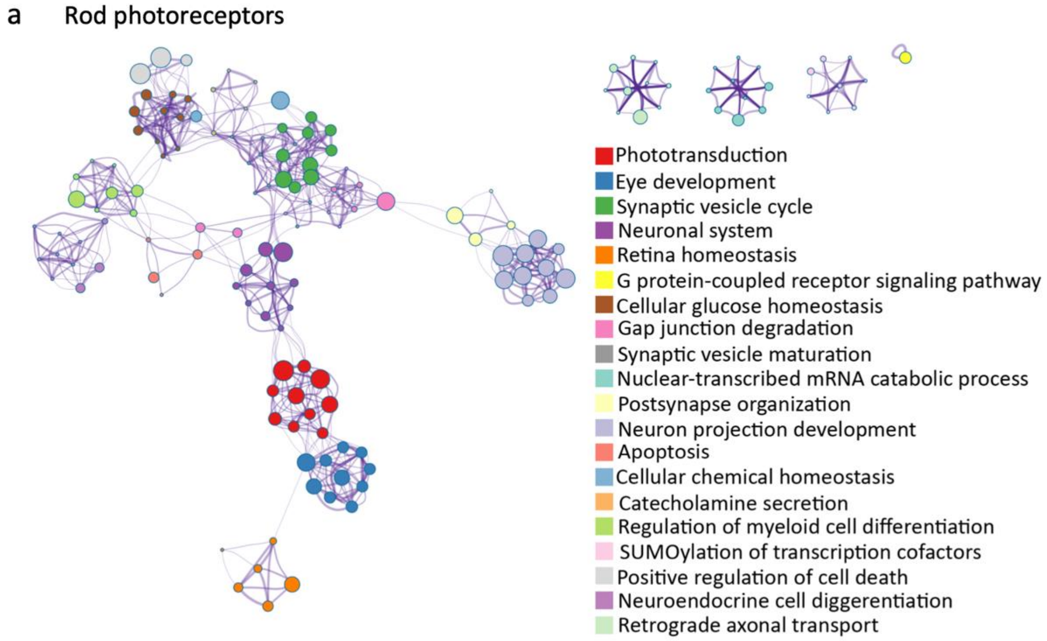

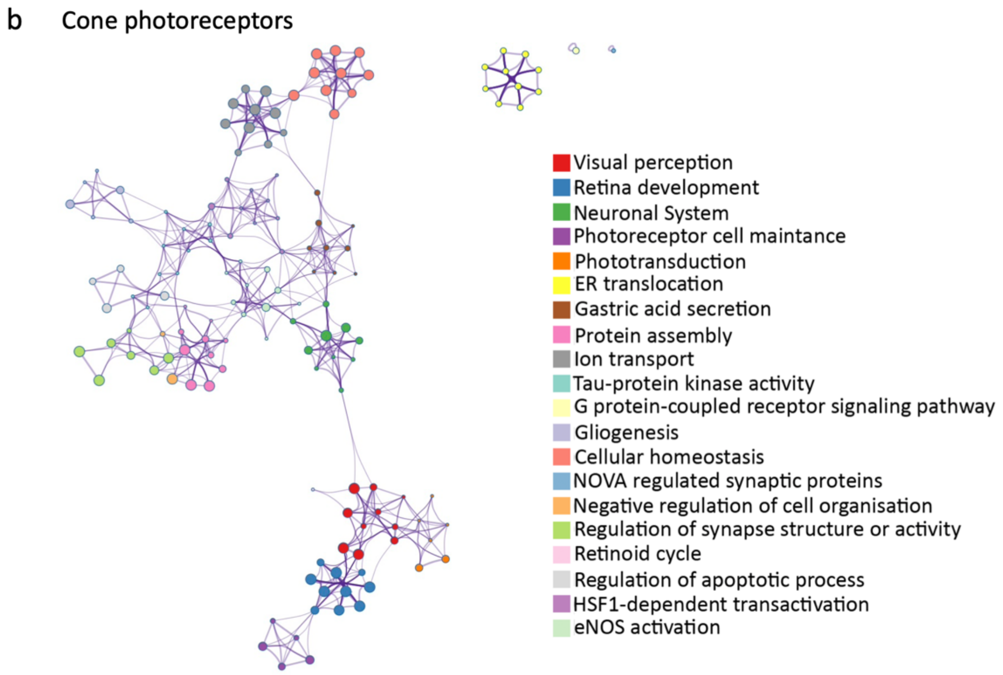

Figure S2. Metascape network analysis on rod (a) and cone photoreceptors (b). Metascape biological network analysis with each term represented by a circle node, size proportional to the number of input genes falling under that term, and color representing cluster identity. Terms with a similarity score > 0.3 are linked by an edge. Term labels were only shown for one term per cluster for clarity.

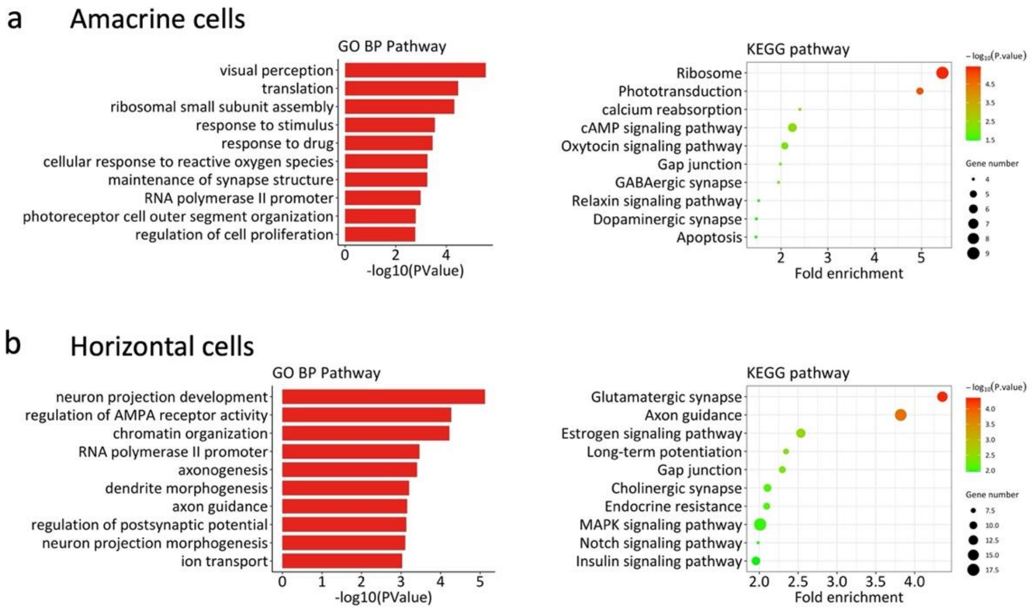

Figure S3. Pathway analysis on amacrine (a) and horizontal cells (b) at post-natal day (P)13. Data showing differentially enriched genes (DEGs) from *rd1* compared to wild-type (WT). Left panel: Gene ontology (GO) biological category (BP) analysis of the DEGs showing the top 10 most enriched GO BP terms. X-axis indicates statistical significance of differential expression as -log10 (p-value). Y-axis shows GO BP categories. Right panel: scatter plot of DEGs KEGG enrichment. X-axis indicates fold enrichment. Y-axis specifies KEGG pathways. Dot size indicates number of DEGs per pathway. Color coding indicates p-value range.

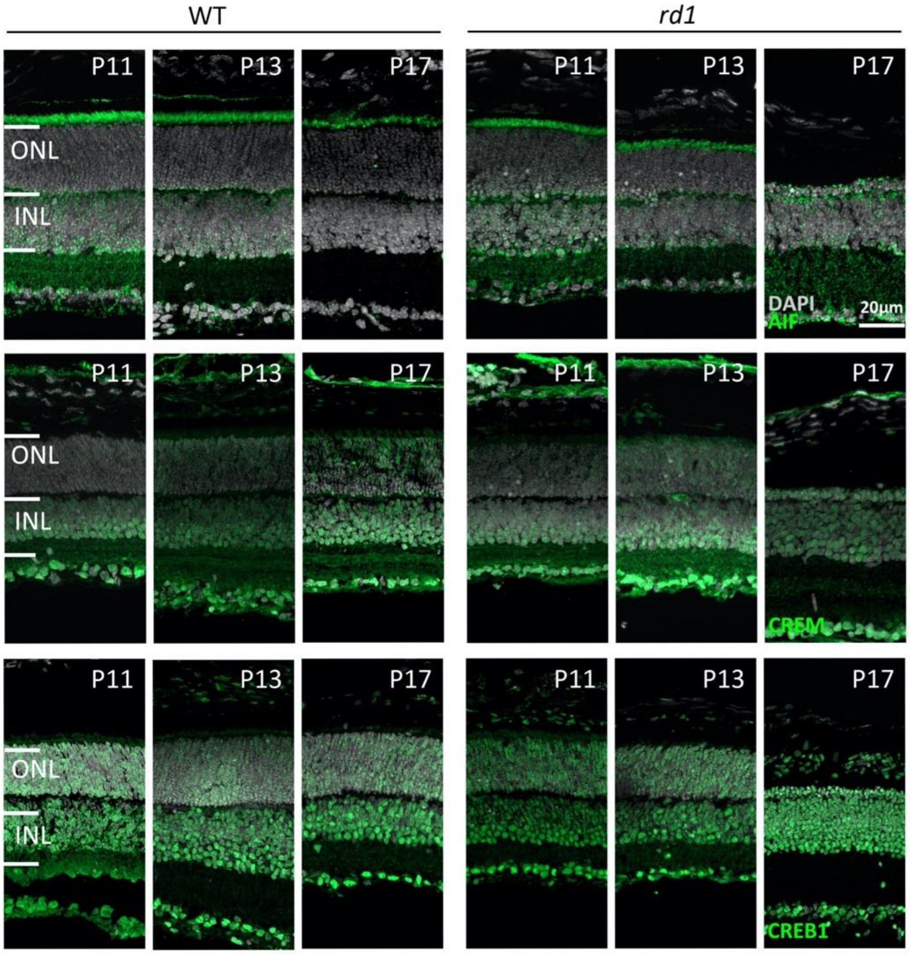

Figure S4. Retinal expression of AIF, CREM, and CREB1 during the 2nd post-natal week. (**a**) AIF labelling of photoreceptor inner segments was mostly lost in P17 *rd1* retina. (**b**) IF for CREM labelled the entire retina, without obvious differences between WT and *rd1*. (**c**) Similarly, CREB1 labelling was seen throughout retina without apparent differences between WT and *rd1*. DAPI (grey) was employed as nuclear counterstain. ONL, outer nuclear layer; INL, inner nuclear layer; WT, wild-type; *rd1*, retinal degeneration 1.

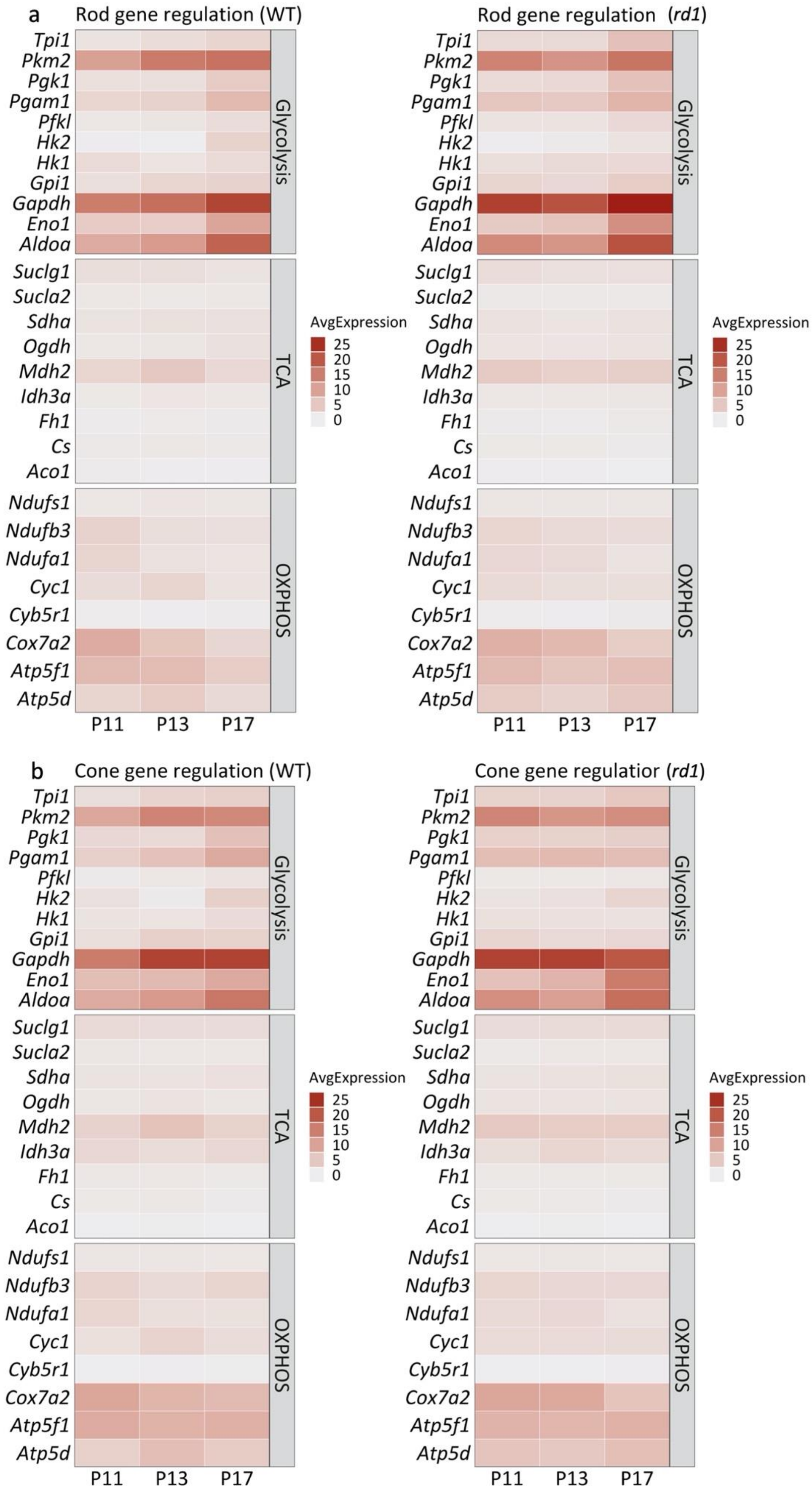

Figure S5. Normalized average expression of in individual genes involved in glycolysis, TCA cycle, and OXPHOS. (**a**) Normalized average expression of rod genes involved in glycolysis, TCA cycle, and

OXPHOS. (**b**) Normalized average expression of cone genes involved in glycolysis, TCA cycle, and OXPHOS.

Supplementary dataset S1: This dataset is an analysis of variance for comparing statistical differences in gene expression between cell populations of interest, which includes rods, cones, Müller glia cells, amacrine cells, and horizontal cells. We use the Seurat and FindMarkers function of the R package for variance analysis to compare between wild-type and *rd1* retinas. The screening threshold for differential genes was $|avg\_log_2FC| > 0.58$ and $p\_val < 0.05$. In dataset, feature: gene name. p_val: p-value of significance of differential expression. avg_log₂FC: log 2-fold change of the average expression value of the two cell populations. Here, $log_2FC = log_2(rd1) - log_2(wt)$. pct.1: the percentage of cells expressing the gene in the cell population of the experimental group. pct.2: percentage of cells expressing the gene in the cell population of the control group. p_val_adj: adjusted p-value. geneDescription: description of the gene.

Table S1. The numbers and percentages of cells identified per cell type and time-point.

celltype	WT_P11	WT_P11.perc(%)	WT_P13	WT_P13.perc(%)	WT_P17	WT_P17.perc(%)	Rd_P11	Rd1_P11.perc(%)	Rd1_P13	Rd1_P13.perc(%)	Rd1_P17	Rd1_P17.perc(%)
Rod	4241	82.48	6908	54.24	4213	81.19	4239	54.71	4532	63.11	950	15.88
Muller	198	3.85	1371	10.76	301	5.8	891	11.5	475	6.61	797	13.32
Bipolar	498	9.68	3922	30.79	461	8.88	2075	26.78	1167	16.25	2715	45.39
Amacrine	117	2.28	292	2.29	80	1.54	183	2.36	280	3.9	837	13.99
Cone	78	1.52	197	1.55	108	2.08	259	3.34	303	4.22	571	9.55
Horizontal	4	0.08	11	0.09	7	0.13	5	0.06	318	4.43	27	0.45
Microglial	5	0.1	25	0.2	6	0.12	26	0.34	75	1.04	72	1.2
Vascular	1	0.02	11	0.09	13	0.25	70	0.9	31	0.43	13	0.22
